# Study Protocol of the Ten Years Up Project: Mapping the Development of Self-Regulation Strategies in Young Adults Over Time

**DOI:** 10.3389/fpsyg.2021.729609

**Published:** 2021-09-17

**Authors:** Marleen Gillebaart, Jantina Brummelman, Denise de Ridder

**Affiliations:** Social, Health, and Organisational Psychology, Utrecht University, Utrecht, Netherlands

**Keywords:** self-regulation, personal goals, young adults, prospective cohort, well-being

## Abstract

Self-regulation is an important predictor of many outcomes relating to health and well-being. Research thus far has not systematically addressed the development of self-regulation strategies during young adulthood, but instead has focused on the predictive value of childhood self-regulation competence for outcomes later in life. The present study protocol describes the Ten Years Up (10YUP) project, a longitudinal cohort of young adults who will be followed for Ten years. By adopting a dynamic approach, we aim to examine how the nature and frequency of self-regulation strategies develop over time, document to what extent the use of strategies is affected by contextual and personal factors, and determine how these strategies affect health and well-being over the course of ten years. The 10YUP project employs a prospective longitudinal design to map the development of self-regulation strategies over time. A sample of 3,000 participants will be recruited by random selection from the general population of 16-year olds to retain a final sample of 1,000 participants after Ten years (accounting for an estimated drop-out rate of 10% each year). A mobile app will be used to collect data every 3 months. Self-regulation strategies will be assessed by means of the *Goal Setting and Striving Inventory* that asks participants to list their personal goals and then choose their most important goal to answer items about goal perception and strategy use. The resulting composite self-regulation index will be related to a wide range of contextual and personal factors that may act as either antecedents or consequences of self-regulation, depending on their specific time of assessment (either prior to or following self-regulation assessment) by means of cross-lagged panel analyses and other analyses allowing for establishing causal relationships over time. The 10YUP project is likely to generate novel insights into the development of self-regulation in young adulthood, how this development is affected by personal and contextual factors, and how these in turn may be influenced by how young people self-regulate—which is important for public policies aimed at guiding young people's choices and how they affect their health and well-being.

## Introduction

Self-regulation, defined as the ability to set personal goals, determine and follow through on strategies to achieve them, and regulate emotions when encountering obstacles and setbacks, is known to be an important predictor of many life outcomes in the domain of health and well-being (Tangney et al., 2004; Moffitt et al., 2011). Self-regulation is related to happiness and life satisfaction (Cheung et al., 2014; Hofmann et al., 2014), higher quality of interpersonal relationships (Vohs et al., 2011), better academic performance (Tangney et al., 2004; Duckworth and Seligman, 2005), and less financial debt, unemployment, obesity, and substance abuse (Tangney et al., 2004; De Ridder et al., 2012). During young adulthood (16–25 years of age), self-regulation is particularly important because in this phase in life individuals increasingly have to make their own decisions and set their own goals (e.g., regarding school, work, relationships, health, and finances) that may have consequences for the rest of their lives. Research into youngster's self-regulation has focused on self-regulation competence in childhood, and its predictive value for outcomes later in life (Mischel et al., 1988; Moffitt et al., 2011). However, research thus far has not systematically addressed the employment or development of self-regulation strategies during young adulthood in a more dynamic way. Gaining insight regarding the underlying mechanisms of self-regulation during young adulthood at a population level can inform effective health and well-being policies. The Ten Years Up (10YUP) project therefore highlights self-regulation in young adulthood as a crucial factor in understanding how young people regulate their lives with regard to important decisions in many important areas over time.

The present study protocol paper describes the design of the 10YUP project, which is set up to examine underlying mechanisms and dynamics of self-regulation in a representative young adult community sample over the course of Ten years. Importantly, the 10YUP design allows for addressing three critical gaps in the knowledge regarding self-regulation. First, we will address changes in self-regulation specifically during the period of young adulthood rather than focusing on fixed self-regulation competence in childhood or adulthood. Second, we will examine a wide variety of self-regulation strategies rather than the mere inhibition of undesired responses (i.e., not doing things that are bad for you such as poor school performance, engaging in aggressive, and/or addictive behaviors), which has been the classic focus of self-regulation research. Third, this study is one of the first to adopt a dynamic prospective approach, in which self-regulation and its determinants and outcomes are assumed to change and influence each other over time. We will review each of these critical gaps below.

The period of young adulthood is a significant stage during which the impact of self-regulation at one point in time may have key consequences later in life. Young adulthood is characterized by increased independency from parents or other adult caretakers, in which young people have to make their own decisions and set their own goals regarding relationships, social networks, school, work, personal finance and many more (Koepke and Denissen, 2012). Surprisingly, research specifically addressing the use and development of self-regulation strategies during young adulthood is thus far scarce. Instead, self-regulation has been considered a fixed competence that is formed during early childhood and determines outcomes (much) later in life. For example, it has been shown that performance on a delay of gratification task in preschool children predicted cognitive, social and academic competence and ability to deal with frustration and stress ~ 11–14 years later (Mischel et al., 1988; Shoda et al., 1990). Furthermore, Moffitt et al. (2011) showed that childhood self-regulation (measured at 5 years of age) could predict physical health, substance dependence, personal finance, and criminal conviction thirty years later. However, there is also evidence suggesting that self-regulation is not a static competence formed in early childhood, but more fluid and developing over time under the influence of different circumstances in one's life (Gestsdóttir and Lerner, 2007, 2008; Farley and Kim-Spoon, 2014). To illustrate, young people become more self-aware and reflective of their own mental processes when growing into adulthood, which enables them to improve their reasoning and planning behavior and modify their environment to achieve their goals (e.g., Bakracevic Vukman, 2005; Demetriou and Bakracevic, 2009). Furthermore, more accurate evaluation of one's own performance with respect to goal pursuit might lead to a rearrangement of previously used self-regulation strategies in order to obtain desired outcomes (e.g., Mischel et al., 1989). However, currently, direct examination of self-regulation dynamics focused on young adulthood is lacking. The present study therefore aims to fill this gap in knowledge by examining the nature, frequency, and dynamics of self-regulation strategies in this age group.

For a long time, the ability to override predominant undesirable response tendencies (i.e., interfering with the pursuit of long-term goals) has been regarded the core ingredient of self-regulation. Classical studies on delay of gratification in 4 year old children attest to this approach (Mischel et al., 1988, 1989). The central role of inhibition in self-regulation has also been demonstrated in adolescents (e.g., Wulfert et al., 2002; Wills et al., 2007). However, inhibiting a response (e.g., resisting the urge to go out with friends is usually not sufficient to achieve a long-term goal [e.g., academic performance)]. One also has to initiate congruent behavior in order to achieve this goal (e.g., studying) (De Ridder et al., 2011; De Ridder and Gillebaart, 2017). Moreover, inhibition of impulses is an effortful process (Fujita, 2011; Gillebaart and de Ridder, 2015; De Ridder and Gillebaart, 2017; Kroese, 2019) which may be subject to fatigue (Baumeister et al., 1998; Vohs et al., 2008). Therefore, self-regulation strategies other than inhibition are important during goal pursuit (Mann et al., 2013; Adriaanse et al., 2014; De Ridder and Gillebaart, 2017).

A new perspective on a broader variety of self-regulation strategies emerged recently, suggesting a range of self-regulation strategies beyond effortful inhibition. Different models of these strategies have been proposed. For instance, Duckworth et al. (2016) presented the “process model of self-regulation,” in which self-regulation strategies are organized through a cycle of four strategies: situation selection, attentional deployment, cognitive reappraisal and response modulation. Indeed, it has been shown that seeking distraction from undesired responses and cognitive reappraisal in order to diminish undesired responses and amplify desired responses by thinking differently about a certain situation leads to increased subjective well-being, while inhibition has shown a negative relationship with well-being (Nielsen et al., 2019). Furthermore, a model has been proposed in which effortless self-regulation strategies (e.g., habitually avoiding or down-regulating undesired responses) are a key ingredient for successful self-regulation and goal pursuit. In addition to the initiation of desired behavior, these type of strategies are key components of self-regulation in the experience of well-being (Gillebaart and de Ridder, 2015; De Ridder and Gillebaart, 2017). In line with this notion, it has been shown that higher levels of trait self-control are associated with a stronger focus on positive consequences of a particular challenging task, goal setting, and the employment of emotion regulation strategies (Hennecke et al., 2019). Individuals with high trait self-control are also faster in the identification of self-control conflicts (Gillebaart et al., 2015; Schneider et al., 2019), potentially allowing for avoidance of situations that would challenge their self-regulation (Ent and Baumeister, 2015), and less experience of self-control conflicts (De Ridder et al., 2012; Hofmann et al., 2012). In addition, high trait self-control has been associated with stronger autonomous motivation to pursue goals (Converse et al., 2019) and greater employment of effortless self-regulation strategies such as adaptive habits (Adriaanse et al., 2014; Galla and Duckworth, 2015; Gillebaart and de Ridder, 2015). The present study will build on this new self-regulation perspective by focusing on a broad range of self-regulation strategies.

**Figure 1:**
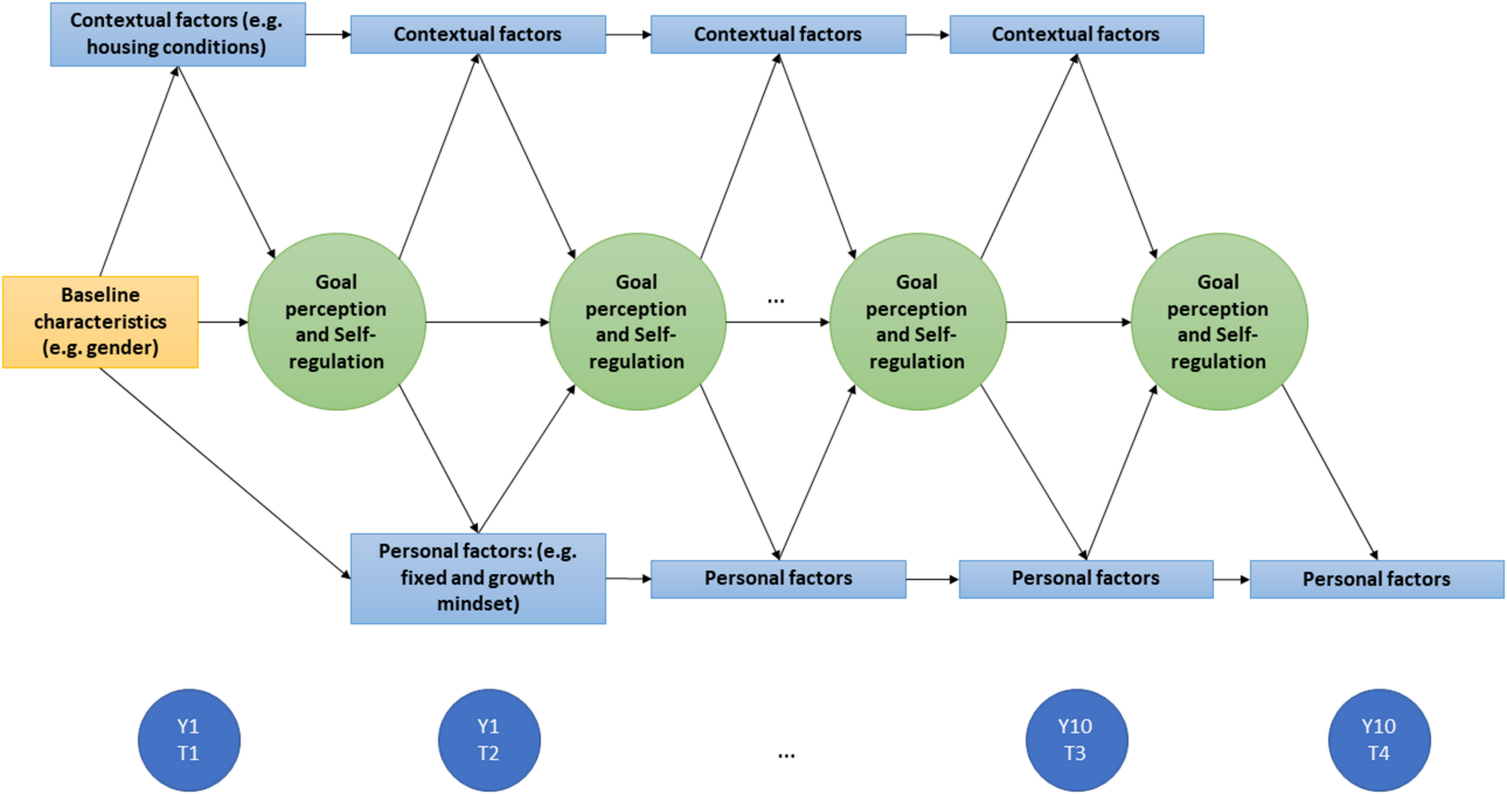
Dynamic model of all factors measured in the 10YUP cohort, showing the expected underlying relationships.

Self-regulation is known to be related to a wide variety of psychological factors (e.g., personality and self-esteem) (Crocker et al., 2006; Denissen et al., 2013), and plays a crucial role in many outcomes related to health and well-being (Tangney et al., 2004; Moffitt et al., 2011). Associations between self-regulation and its determinants and outcomes have thus far mainly been studied cross-sectionally and in relative isolation, with self-regulation considered to be a causal predictor of outcomes. However, outcomes may in turn also affect self-regulation (e.g., life satisfaction may be positively related to self-esteem and self-efficacy, which eventually leads to more successful self-regulation). This focus on specific short-term associations prevents a good understanding of how self-regulation develops and is affected by dynamic reciprocal relationships. Both contextual (e.g., living conditions such as neighborhood characteristics and type of household) and personal (e.g., self-efficacy) factors may fluctuate over time and as such relate to self-regulation changes over time. Studying self-regulation longitudinally therefore requires a dynamic approach that is often employed in prospective psychological research focusing on developmental cascades (e.g., Masten and Cicchetti, 2010). The present project aims to address this knowledge gap in self-regulation research by highlighting the interplay between contextual and personal factors and self-regulation over time, in which determinants may become outcomes depending on their specific time of measurement (see Figure 1).

In summary, the 10YUP project will address critical gaps in self-regulation research by adopting a prospective dynamic approach in which self-regulation strategies in relation to personal and contextual factors will be examined over the course of young adulthood. As such, the 10YUP project has the following three aims: (1) to examine how the nature and frequency of self-regulation strategies develop over time during the critical period of young adulthood; (2) to document to what extent the use of self-regulation strategies is affected by contextual (e.g., socio economic status) and personal factors (e.g., personality traits, self-esteem); and (3) to determine how these strategies affect health and well-being over the course of time with an emphasis on critical choices that have implications for health and well-being.

## Methods

### Participants and Recruitment

We aim to obtain longitudinal data from 1,000 young adults. Participants will be recruited through random selection from the general population of 16-year-olds as registered by Statistics Netherlands (CBS). This enables us to include a broad population in terms of SES. Since this study requires long-term intensive commitment, it is taken into account that the response rate will be relatively low and that part of the study population will get lost to follow-up during the Ten years of the study. Therefore, an initial sample of 3,000 individuals is required in order to retain 1,000 participants after Ten years, allowing for a drop-out rate of 10% each year. A response rate of 5% is expected. This number is conservatively derived from a recent study in a young Dutch sample with a response rate of 6.1% (Lugtig et al., 2019). Other research has similarly shown that response rates in studies among adolescents (15–19 years of age) are generally lower than in older populations (Hellevik, 2016). Furthermore, response rates have been decreasing in the past few years (De Leeuw and De Heer, 2002). Therefore, sixty thousand young persons (16 years of age at recruitment) from three provinces in the Netherlands, covering both rural and urban areas, will be invited to participate through a postal letter (only residential addresses are available in this database). Besides age, there are no additional eligibility criteria. To increase the initial response rate and to prevent biased response from people who have high self-regulation ability, this letter will be made appealing both through the physical appearance of the letter (e.g., the use of colors and pictures) and the content focusing on different kinds of motives to participate (e.g., contribution to science, self-understanding, to help the researchers, and the possibility to win prizes. The initial sample size of 3000 will allow us to study differences between young persons (in terms of socio-economic as well as other background characteristics), and study a variety of trajectories in terms of school and work for people between ages 16 and 25.

### Study Design

The present study comprises a prospective longitudinal design, in which the dynamic interplay between self-regulation strategies and personal and contextual factors will be examined over the course of Ten years, with the project kicking off in 2020. Personal and contextual factors that are considered to be relatively stable and that are less central to the study's main question will be assessed at baseline, while factors that are expected to change over time will be assessed more frequently (at least once a year).

### Procedure

**Figure 2:**
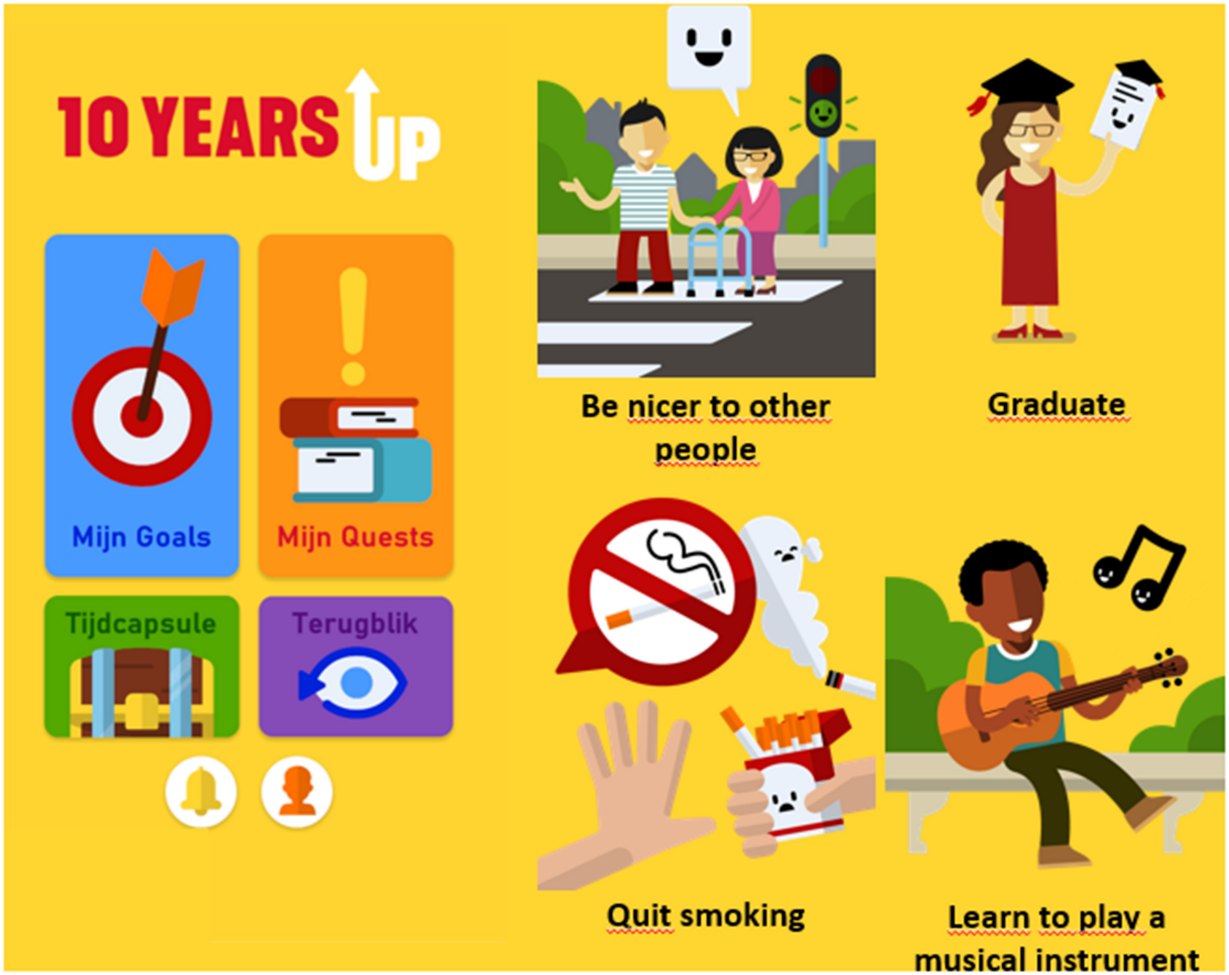
Impression of the 10YUP app. The home screen is shown on the left hand side and includes different modules to fill in the GSSI (“My Goals”), fill in the other questionnaires (“My Quests”), and two buttons which are intended to provide incentives to participants (treasure chest). On the right hand side, four examples of goals from the GSSI are presented. Source: 10YUP app; Copyright: © 10YUP.

All data will be collected using an in-house developed mobile application (10YUP app) that is suitable for both Android and Apple users (Figure 2). Participants will download the 10YUP app at the start of the study. For people interested in study participation who do not possess a smart phone, a limited number of phones will be made available. Upon first participation, they will read and sign informed consent and provide their contact information (name, e-mail address, and phone number) within the 10YUP app. Contact information will be stored separately from other data in accordance with privacy regulations. Collected data will be pseudonymized using a participant identification number while the study is running.

Over the course of Ten years, participants will be invited every 3 months through push messages within the app as well as via e-mail to complete a survey via the 10YUP app. A schematic overview of the measurements per time-point in the first year is presented in Table 1. Data collection will consist of three parts: (1) self-regulation, comprising the assessment of personal goals, goal perception, and self-regulation strategies; (2) contextual factors (e.g., SES, living conditions); and (3) personal factors, including psychological and behavioral assessments. Personal and contextual factors may serve as predictor or outcome of self-regulation, depending on time of measurement (e.g., a psychological factor can be an outcome of self-regulation earlier in the process, but the same psychological factor can predict self-regulation outcomes later on), as is illustrated in Figure 1. In addition, there will be room for smaller quantitative and qualitative studies on subsets of the sample to cross-validate findings, generate ideas, and enrich data.

**Table 1:** Schematic overview of the measurements per time-point for the first year of the project.

	**Timepoint 1**	**Timepoint 2**	**Timepoint 3**	**Timepoint 4**
Baseline assessments	• Age • Gender		• Ethnic background • Parental level of education	-
Contextual factors	• Level of education • Social support	• Housing condition • Religion • Socio Economic Status (SES)	-	Life events
Personal factors	• Happiness • School/work performance • Self-esteem • Sleep	• Big five personality • Consideration of future consequences • Exercise • Financial behavior • Future self-continuity • Nutrition	• Adolescent self-regulatory inventory (ASRI) • Criminality and aggressive behavior • Fixed/growth mindset • General well-being • Sustainable behavior	• Alcohol use • Bullying • Drug use • Life satisfaction • Screen use • Sense of Mastery • Smoking • Trait self-control
Self-regulation measurement	Goal Setting and Striving Inventory (extended version) (GSSI)	GSSI (short version)	GSSI (short version)	GSSI (short version)

*Data will be collected every 3 months*.

Participants who drop out will be approached via a text (SMS) and an accompanying phone call in which they are asked about their reasons to (not) participate. This will allow for retention of participants who are motivated to be part of the study but have dropped out for other reasons (e.g., forgetting), as well as gathering information from participants who deliberately dropped out.

#### Incentives

Adolescents will not be financially reimbursed for study participation. Rather, based on pilot studies we expect participants to remain in the study because of a genuine interest in their future and whether they will be able to reach the goals that they have set for themselves. To promote intrinsic motivation for study participation, a personal “treasure chest” (time capsule) has been developed that allows participants to store their personal goals for a dedicated time and that is available only for themselves. This chest can also be used for writing to their future selves or store other personal documents that relate to their future (such as picture, poems, or diaries). This treasure chest will not be accessible by the researchers. We will regularly provide participants with feedback on information obtained from the surveys (e.g., what kind of goals are frequently adopted) and study progress in general by speaking to their motives for participating (e.g., a general interest in science or wanting to know more about self-regulation). Finally, small material incentives such as gift cards or vouchers will be used to promote study adherence.

### Measurements

#### Self-Regulation Strategies

One of the main aims of the 10YUP project is to gain better insight into self-regulation strategies employed by young adults. To this end, a new scale has been developed: the *Goal Setting and Striving Inventory* (GSSI; see Supplementary Materials). The GSSI consists of three parts. In the first part, participants indicate which goals currently apply to them from a list of 35 goals that were derived from a series of studies with young adults, including focus groups, online questionnaires, and qualitative interviews. These goals comprise a wide variety of strivings, ranging from losing weight to taking the bike more often, from improving school grades to using less plastic, and from saving money for a holiday to improving one's gaming performance. Focus group findings have revealed that goals names by adolescents may be either short or long term, concrete or abstract, and related to either approaching a desired outcome or avoiding an undesired outcome. Participants are instructed to swipe each of the 35 goals to the left-hand side of the screen if the goal does not apply to them, or to the right-hand side of the screen if it does apply. All goals relevant to the participant are rated on the dimensions of importance and feasibility on a Visual Analogue Scale (VAS) ranging from 0 (not important/feasible at all) to 100 (extremely important/feasible). Participants have the opportunity to add a personal goal that is not included in the list and additionally indicate its importance and feasibility.

In the second part, participants will select their most important goal from the list of goals relevant to them. This goal will consequently serve as a target to assess goal perceptions, including self-efficacy, locus of control, autonomous/controlled motivation, task aversion, and prevention/promotion focus, on a VAS ranging from 0 (not applicable at all)−100 (completely applicable). In the third part of the GSSI, participants' self-regulation strategies will be assessed while keeping the selected goal in mind. They will be asked to what extent they use different strategies to achieve their previously selected goal: planning, monitoring, seeking social support, situation selection, cognitive reappraisal, automatization, perseverance, inhibition, initiation and rewarding. Individuals again provide their answer on a VAS ranging from 0 (not applicable at all)−100 (completely applicable). The complete version of the GSSI will be assessed at T1 of each year. On T2, T3, and T4 of each year, a shortened version will be administered, in which participants complete the second and third part of the GSSI.

Next to documenting goal perceptions and self-regulatory strategies for the selected (idiosyncratic) goal, all participants will answer questions about one goal that is similar to all participants throughout the whole period of ten years (“being successful in school/work performance”). This reference goal allows for directly comparing strategies over time and across participants.

#### Personal and Contextual Factors

A large number of personal and contextual factors that are expected to be related to self-regulation will be assessed (see Table 1 and Additional file 1 for an overview).

##### Baseline Assessments

Demographic information (including gender, year and month of birth, parental level of education, and ethnic background) will be recorded at baseline.

##### Personal and Contextual Factors

The following concepts will be (repeatedly) assessed throughout the course of the study: adolescent self-regulation, Big Five personality, consideration of future consequences, fixed and growth mindset, future self-continuity, housing conditions, level of education, life events, religion, self-esteem, sense of mastery, SES, social support, and trait self-control. Furthermore, we will assess alcohol use, bullying, criminality and aggressive behavior, drug use, exercise, financial behavior, general well-being, happiness, life satisfaction, school and work performance, screen use, sleep, smoking, and sustainable behavior. These concepts will give insight into someone's psychological make-up and/or behavior relevant for self-regulation and will be assessed once a year to determine their development and their associations with self-regulation, either as a determinant or an outcome.

### Data Analysis

The large sample size and the repeated assessment of central constructs allows for examining both differences between (groups of) young adults (e.g., using analyses of variance with personal and contextual factors as outcomes) and change over time, both within persons and for the cohort as a whole. In conjunction with the three main study objectives, the following data analytic procedures will be employed. First, the development of self-regulation strategies over time will be determined by (multilevel) growth curve analysis based on regression principles using the self-regulation strategy measurements as input. This will reveal whether young adults use different strategies over time and whether strategy use is dependent on the type of goal and goal perception. Additionally, cross-lagged panel models (for the first series of measures) and multilevel regression models will be applied in order to examine the influence of personal and contextual factors (e.g., fixed and growth mindset, level of education, life events) on the use of self-regulation strategies (e.g., monitoring, automatization), and the predictive value of self-regulation strategies on these factors in a gradient-like fashion rather than using cut-off points. This will be done both cross-sectionally (using a single timepoint) and over time (using multiple timepoints).

## Discussion

The present paper has described the protocol of the 10YUP project that will examine the development of self-regulation and its dynamic interplay with a wide variety of personal and contextual factors over the course of Ten years in a large sample of young adults. This approach allows for addressing three critical gaps in knowledge regarding self-regulation in young people. Whilst the whole project has been carefully planned, we anticipate a number of foreseeable challenges. First, based on previous prospective research in adolescents, we expect low response to our initial invitation and relatively high drop-out over the course of the study, which may lead to bias due to self-selection. Nevertheless, our recruitment strategy comprising the invitation of a large random selection from the general population of 16-year-olds does allow for reaching out to segments of the population that are hard to reach. A second challenge will be to keep participants committed and willing to participate once they are included. To this end, we will stimulate intrinsic motivation for participation by providing the possibility to store their personal within a “treasure chest” that is accessible only for themselves. Third, our design does not allow for comparison with a control group that does not participate in the project. As such, we cannot definitively exclude influence of our measurements on the sample's self-regulation dynamics. However, drop-out analyses will provide some insight into possible changes between those who do and do not participate. Finally, a challenge of the ambitious 10YUP project is to connect insights into the development or individual self-regulation to participants' contextual living conditions and translate these connections into a body of knowledge that is relevant for public policies aimed at increasing young people's health and well-being with a particular focus on increasing control over their lives. As such, the 10YUP project aims to contribute to a more profound understanding of how young people's choices have an impact on their own lives as well as societal challenges that depend on individual decisions.

In conclusion, the 10YUP project will use a dynamic and longitudinal approach to provide novel insights into the development of self-regulation in young adulthood, how this development is affected by personal background and their living conditions, and how these in turn may be influenced by how young people self-regulate. We expect our project to generate unique knowledge into how young people perceive, pursue, and achieve their personal goals during a critical period of growing up.

## Ethics and Dissemination

Ethical approval for all study procedures was granted by the Faculty Ethics Review Committee (FETC) of the Faculty of Social Sciences at Utrecht University (filed under number 19-124). In addition, a Data Protection Impact Assessment (DPIA) was made in order to safeguard privacy requirements regarding data collection and storage. The DPIA was approved by the Privacy Officer at Utrecht University. All study procedures are designed and performed in accordance with the Declaration of Helsinki (World Medical Association, 2018).

Dissemination of the project's finding comprise the following. The 10YUP project is designed not only to examine self-regulation by the researchers directly involved in the study, but also to provide other researchers with the possibility to conduct secondary analyses. Once the cohort has been assembled, a data repository will be set up to accommodate other researchers. A Data Management Protocol has been set up that specifies detailed procedures for data collection, storage, retrieval, and sharing according to the FAIR principle (i.e., making data findable, accessible, interoperable and reusable). Applications for inclusion of instruments in future waves of the cohort will be considered by the study team and an independent nationwide advisory board of experts in the field. Data collection and data storage will adhere to the guidelines of Utrecht University that are in accordance with (inter)national regulations. Completed data waves will be made available annually at the repository of the Dutch National Centre of Expertise and Repository for Research Data (dans.knaw.nl), hosted by the Royal Netherlands Academy of Arts and Sciences.

## Ten Years Up Consortium

Jeroen Benjamins^1,2^, Jaap Denissen^3^, Floor Kroese^1^, Peter Lugtig^4^, Jet Smit^5^, and Jan Fekke Ybema^1^ (in alphabetical order)

^1^Department of Social, Health and Organisational Psychology, Utrecht University, Utrecht, Netherlands

^2^Department of Experimental Psychology, Helmholtz Institute, Utrecht University, Utrecht, Netherlands

^3^Department of Developmental Psychology, Utrecht University, Utrecht, Netherlands

^4^Department of Methodology and Statistics, Utrecht University, Utrecht, Netherlands

^5^Department of Public Health, Healthcare Innovation & Evaluation and Medical Humanities, University Medical Centre Utrecht, Julius Center, Utrecht, Netherlands.

## Author Contributions

MG, DdR, and JB wrote the protocol. All authors were involved in setting up the Ten Years Up study that is described in the protocol.

## Funding

The Ten Years Up project was funded by ZonMw Grant No. 91118003. ZonMw was not involved in the design of the study protocol.

## Conflict of Interest

The authors declare that the research was conducted in the absence of any commercial or financial relationships that could be construed as a potential conflict of interest.

## Publisher's Note

All claims expressed in this article are solely those of the authors and do not necessarily represent those of their affiliated organizations, or those of the publisher, the editors and the reviewers. Any product that may be evaluated in this article, or claim that may be made by its manufacturer, is not guaranteed or endorsed by the publisher.
