# Supplementary material for: Study Protocol of the Ten Years Up Project: Mapping the Development of Self-Regulation Strategies in Young Adults Over Time
Source: Front Psychol. 2021 Sep 17;12:729609. doi: 10.3389/fpsyg.2021.729609 (PMC8486174; doi:10.3389/fpsyg.2021.729609)
Supplement: Supplementary file 1 [file Data_Sheet_1.PDF]

Goal Setting and Striving Inventory

Part 1: List of goals

Saving money for a long holiday
Go to bed in time more often
Less screen time
Exercise more often
Finish my homework in time
Get to school/work in time
Quit smoking
Tidy up my room more often
Lose weight
Being more kind to other people
Reduce my alcohol intake
Reduce single-use plastic
Reduce meat consumption
Graduate from high school
Walk or cycle more often
Earn my own money
Increase fruit and vegetable consumption
Pay off financial debts
Reduce consumption of energy drinks
Decide about next steps of my education
Have a better relationship with my parents
Live on my own
Learn to play a music instrument
Becoming successful

Meet with my friends more often

Improving my sports performance

Earn a lot of money

Enjoy life more

Get my driver's license

Learn another language

Experience less stress

Become more self-confident

Find a job

Part 2: Goal Perceptions

1 I am uncertain whether I can achieve this goal (Self-Efficacy) *

2 It is a matter of luck whether I will achieve this goal (Internal Locus of Control) *

3 I aim for this goal because other people expect me to (Controlled Motivation)

4 This is a goal that I truly desire (Autonomous Motivation)

5 I dislike the things I need to do to achieve this goal (Task Aversion)

6 Achieving this goal will put me at ease (Prevention Focus)

7 Achieving this goal will make me happy and proud (Promotion Focus)

*Reverse coding

Part 3: Self-Regulatory Strategies

1 I make a plan of action (Planning)

2 I check whether I am on track (Self-Monitoring)

3 I ask other people for support (Social Support)

4 I am looking for opportunities (Situation Selection)

5 I stay positive when I make little progress (Cognitive Reappraisal)
6 I make a routine of things I need to do (Routine Building)
7 I persist when I encounter difficulties (Persistence)
8 I suppress the impulse of giving up (Self-Control)
9 I know what I need to do but I keep postponing (Initiation) *
10 I reward myself when I make progress (Rewarding Oneself)

*Reverse coding
